# Supplementary material for: Effect of Linear and Nonlinear Pedagogy Physical Education Interventions on Children’s Physical Activity: A Cluster Randomized Controlled Trial (SAMPLE-PE)
Source: Children (Basel). 2021 Jan 15;8(1):49. doi: 10.3390/children8010049 (PMC7830495; doi:10.3390/children8010049)
Supplement: Supplementary file 1 [file children-08-00049-s001.zip › children-1040334-supplementary/children-1040334-Supplementary material Table S2 - Reasons for missing physical activity data.docx]

**Table S2.** Reasons for missing physical activity data.

|  | **Linear Pedagogy** | **Nonlinear Pedagogy** | **Control** |
| --- | --- | --- | --- |
|  | ***N* children = 105** | ***N* children = 112** | ***N* children = 143** |
| **Baseline** |  |  |  |
| Child was absent | 6 | 6 | 4 |
| Child lost the accelerometer | 1 |  | 3 |
| Child did not want to wear an accelerometer | 1 | 3 | 1 |
| Did not meet valid wear time inclusion criteria | 10 | 23 | 40 |
| Valid physical activity observation | 87 | 80 | 95 |
| **Post-intervention** | |  |  |
| Child was absent | 3 | 1 | 1 |
| Child lost the accelerometer | 2 | 3 | 4 |
| Child moved to another school | 1 | 2 | 5 |
| Child did not want to wear an accelerometer | 2 | 3 | 2 |
| Did not meet valid wear time inclusion criteria | 34 | 28 | 60 |
| Valid physical activity observation | 63 | 75 | 71 |
| **Follow-up** | |  |  |
| Child was absent | 4 | 4 | 2 |
| Child lost the accelerometer | 2 | 3 |  |
| Accelerometer technical problem | | 1 |  |
| Child moved to another school | 2 | 6 | 19 |
| Child did not want to wear an accelerometer | 1 | 1 | 3 |
| Child did not receive accelerometer a she or she still had to return one | 3 | 4 | 5 |
| Did not meet valid wear time inclusion criteria | 31 | 33 | 55 |
| Valid physical activity observation | 62 | 60 | 59 |
