# Supplementary material for: Effect of Linear and Nonlinear Pedagogy Physical Education Interventions on Children’s Physical Activity: A Cluster Randomized Controlled Trial (SAMPLE-PE)
Source: Children (Basel). 2021 Jan 15;8(1):49. doi: 10.3390/children8010049 (PMC7830495; doi:10.3390/children8010049)
Supplement: Supplementary file 1 [file children-08-00049-s001.zip › children-1040334-supplementary/children-1040334-Supplementary Material Table S3 - Descriptive data for each time point.docx]

**Table S3.** Descriptive data for each time point.

| **Baseline Data** | | | | | | | | | |
| --- | --- | --- | --- | --- | --- | --- | --- | --- | --- |
|  | **Control (143 children)** | | | **Nonlinear Pedagogy**  **(112 Children)** | | | **Linear Pedagogy (105 children)** | |  |
| **Variables** | **Mean / Number** | **SD** | **Missing data** | **Mean / Number** | **SD** | **Missing data** | **Mean / Number** | **SD** | **Missing data** |
| Sex (Females) | 83 |  | 0 | 58 |  | 0 | 56 |  | 0 |
| Decimal Age (years) | 5.94 | 0.29 | 2 | 5.92 | 0.30 | 1 | 5.95 | 0.30 | 5 |
| White British | 69 |  | 5 | 54 |  | 9 | 66 |  | 8 |
| SEN (Special Educational Needs) | 17 |  | 0 | 17 |  | 1 | 8 |  | 1 |
| IMD Deprivation Decile (arbitrary units) | 1.73 | 1.51 | 3 | 2.52 | 2.05 | 1 | 1.43 | 1.20 | 4 |
| IOTF SDS BMI (arbitrary units) | 0.33 | 1.08 | 28 | 0.51 | 1.11 | 8 | 0.43 | 1.34 | 9 |
| Participation in school sport events | 0 |  | 0 | 0 |  | 0 | 0 |  | 0 |
| Whole week valid hours (hours) | 16.36 | 0.94 | 48 | 16.18 | 1.17 | 32 | 16.34 | 1.11 | 18 |
| Whole week MVPA (minutes) | 68.08 | 18.51 | 48 | 68.84 | 20.31 | 32 | 69.33 | 19.37 | 18 |
| Whole week Mean ENMO (milligravity) | 60.41 | 13.21 | 48 | 60.31 | 14.30 | 32 | 61.88 | 14.58 | 18 |
| Whole week M60 (milligravity) | 214.36 | 66.80 | 48 | 206.27 | 67.70 | 32 | 219.13 | 81.67 | 18 |
| Weekend valid hours (hours) | 16.15 | 1.59 | 48 | 16.11 | 1.64 | 32 | 16.08 | 1.70 | 18 |
| Weekend MVPA (minutes) | 63.18 | 27.76 | 48 | 63.21 | 27.09 | 32 | 65.14 | 28.30 | 18 |
| Weekend Mean ENMO (milligravity) | 55.08 | 20.51 | 48 | 54.07 | 19.060 | 32 | 57.36 | 20.95 | 18 |
| Weekend M60 (milligravity) | 200.65 | 114.07 | 48 | 192.17 | 98.55 | 32 | 217.27 | 142.07 | 18 |
| In school valid hours (hours) | 5.97 | 0.21 | 48 | 5.98 | 0.11 | 32 | 5.95 | 0.29 | 18 |
| In school MVPA (minutes) | 34.26 | 11.72 | 48 | 35.38 | 10.79 | 32 | 31.95 | 9.78 | 18 |
| In school mean ENMO (milligravity) | 89.05 | 24.91 | 48 | 90.62 | 22.80 | 32 | 84.03 | 23.77 | 18 |
| In school M30 (milligravity) | 198.83 | 119.80 | 48 | 206.32 | 114.24 | 32 | 228.61 | 134.24 | 18 |
| Out of school valid hours (hours) | 7.59 | 0.74 | 48 | 7.38 | 0.97 | 32 | 7.60 | 0.74 | 18 |
| Out of school MVPA (minutes) | 28.61 | 9.06 | 48 | 28.32 | 10.43 | 32 | 31.47 | 11.86 | 18 |
| Out of school mean ENMO (milligravity) | 52.48 | 14.40 | 48 | 51.95 | 15.31 | 32 | 57.52 | 19.42 | 18 |
| Out of school M30 (milligravity) | 145.53 | 52.92 | 48 | 146.72 | 56.03 | 32 | 164.50 | 73.87 | 18 |
| Whole week Temperature (Celsius degrees) | 2.62 | 2.10 | 48 | 5.86 | 1.68 | 32 | 5.08 | 0.65 | 18 |
| Whole week Rainfall (mm water) | 1.67 | 1.99 | 48 | 3.53 | 1.50 | 32 | 3.44 | 1.78 | 18 |
| Whole week percentage of daylight (%) | 39.02 | 4.75 | 48 | 35.20 | 1.54 | 32 | 33.71 | 0.75 | 18 |
| During the week Temperature (Celsius degrees) | 2.29 | 2.80 | 48 | 5.67 | 1.03 | 32 | 5.30 | 0.34 | 18 |
| During the week Rainfall (mm water) | 1.46 | 1.69 | 48 | 2.76 | 1.78 | 32 | 1.98 | 2.26 | 18 |
| During the week percentage of daylight (%) | 39.09 | 4.89 | 48 | 35.03 | 1.59 | 32 | 33.52 | 0.75 | 18 |
| Weekend Temperature (Celsius degrees) | 3.16 | 1.18 | 48 | 6.39 | 2.90 | 32 | 4.68 | 1.44 | 18 |
| Weekend Rainfall (mm water) | 2.12 | 2.94 | 48 | 4.71 | 1.86 | 32 | 5.87 | 1.89 | 18 |
| Weekend percentage of daylight (%) | 39.91 | 4.53 | 48 | 35.45 | 1.48 | 32 | 33.99 | 0.77 | 18 |
| ***Meeting guidelines*** |  | % |  |  | % |  |  | % |  |
| Meeting guidelines whole week | 59 | 62.11 | 48 | 51 | 63.75 | 32 | 59 | 67.82 | 18 |
| Meeting guidelines week | 68 | 71.58 | 48 | 57 | 71.25 | 32 | 61 | 70.11 | 18 |
| Meeting guidelines weekend | 50 | 52.63 | 48 | 38 | 47.50 | 32 | 46 | 52.87 | 18 |
| Reaching 30 minutes of MVPA in school | 56 | 58.95 | 48 | 54 | 67.50 | 32 | 48 | 55.17 | 18 |
| Reaching 30 minutes of MVPA outside school | 42 | 44.21 | 48 | 37 | 46.25 | 32 | 47 | 54.02 | 18 |

**Post-Intervention Data**

|  | **Control (143 children)** | | | **Nonlinear Pedagogy**  **(112 Children)** | | | **Linear Pedagogy (105 children)** | |  |
| --- | --- | --- | --- | --- | --- | --- | --- | --- | --- |
| **Variables** | **Mean / Number** | **SD** | **Missing data** | **Mean / Number** | **SD** | **Missing data** | **Mean / Number** | **SD** | **Missing data** |
| Sex (Females) | 83 |  | 0 | 58 |  | 0 | 56 |  | 0 |
| Decimal Age (years) | 6.37 | 0.28 | 2 | 6.34 | 0.30 | 1 | 6.37 | 0.30 | 5 |
| White British | 69 |  | 5 | 54 |  | 9 | 66 |  | 8 |
| SEN (Special Educational Needs) | 17 |  | 0 | 17 |  | 1 | 8 |  | 1 |
| IMD Deprivation Decile (arbitrary units) | 1.73 | 1.51 | 3 | 2.52 | 2.05 | 1 | 1.43 | 1.20 | 4 |
| IOTF SDS BMI (arbitrary units) | -0.01 | 1.37 | 19 | 0.01 | 1.22 | 9 | 0.19 | 1.37 | 6 |
| Participation in school sport events | 0 |  | 0 | 0 |  | 0 | 59 |  | 0 |
| Whole week valid hours (hours) | 15.83 | 1.33 | 72 | 15.93 | 1.19 | 37 | 16.10 | 1.06 | 42 |
| Whole week MVPA (minutes) | 83.69 | 20.86 | 72 | 84.10 | 24.38 | 37 | 89.73 | 27.92 | 42 |
| Whole week Mean ENMO (milligravity) | 73.13 | 17.07 | 72 | 73.84 | 19.32 | 37 | 77.60 | 20.99 | 42 |
| Whole week M60 (milligravity) | 261.53 | 78.65 | 72 | 256.10 | 79.26 | 37 | 280.31 | 85.57 | 42 |
| Weekend valid hours (hours) | 15.57 | 1.77 | 72 | 15.93 | 1.43 | 37 | 16.08 | 1.23 | 42 |
| Weekend MVPA (minutes) | 78.56 | 31.76 | 72 | 78.92 | 31.72 | 37 | 82.84 | 30.70 | 42 |
| Weekend Mean ENMO (milligravity) | 66.31 | 26.11 | 72 | 67.95 | 24.71 | 37 | 68.50 | 21.58 | 42 |
| Weekend M60 (milligravity) | 222.92 | 116.36 | 72 | 239.01 | 100.92 | 37 | 242.87 | 98.44 | 42 |
| In school valid hours (hours) | 5.93 | 0.32 | 72 | 5.96 | 0.16 | 37 | 5.97 | 0.10 | 42 |
| In school MVPA (minutes) | 40.51 | 10.64 | 72 | 40.54 | 11.41 | 37 | 44.37 | 12.31 | 42 |
| In school mean ENMO (milligravity) | 108.42 | 29.72 | 72 | 106.04 | 29.62 | 37 | 115.34 | 28.80 | 42 |
| In school M30 (milligravity) | 231.21 | 126.08 | 72 | 224.71 | 118.23 | 37 | 214.44 | 98.11 | 42 |
| Out of school valid hours (hours) | 7.28 | 1.02 | 72 | 7.23 | 1.04 | 37 | 7.34 | 0.90 | 42 |
| Out of school MVPA (minutes) | 38.28 | 14.96 | 72 | 37.90 | 15.92 | 37 | 39.33 | 20.22 | 42 |
| Out of school mean ENMO (milligravity) | 67.23 | 22.92 | 72 | 68.07 | 26.51 | 37 | 69.80 | 32.17 | 42 |
| Out of school M30 (milligravity) | 249.73 | 94.08 | 72 | 262.70 | 111.32 | 37 | 257.91 | 119.90 | 42 |
| Whole week Temperature (Celsius degrees) | 18.43 | 1.78 | 72 | 18.25 | 1.52 | 37 | 19.04 | 1.77 | 42 |
| Whole week Rainfall (mm water) | 1.07 | 0.80 | 72 | 0.56 | 0.86 | 37 | 1.33 | 0.74 | 42 |
| Whole week percentage of daylight (%) | 70.39 | 0.44 | 72 | 70.50 | 0.45 | 37 | 70.20 | 0.52 | 42 |
| During the week Temperature (Celsius degrees) | 18.81 | 1.81 | 72 | 19.08 | 1.86 | 37 | 18.85 | 1.61 | 42 |
| During the week Rainfall (mm water) | 0.60 | 0.71 | 72 | 0.47 | 0.70 | 37 | 0.72 | 1.08 | 42 |
| During the week percentage of daylight (%) | 70.35 | 0.46 | 72 | 70.48 | 0.44 | 37 | 70.15 | 0.51 | 42 |
| Weekend Temperature (Celsius degrees) | 17.72 | 2.45 | 72 | 16.98 | 2.30 | 37 | 19.35 | 2.29 | 42 |
| Weekend Rainfall (mm water) | 1.86 | 1.63 | 72 | 0.73 | 1.23 | 37 | 2.38 | 1.48 | 42 |
| Weekend percentage of daylight (%) | 70.48 | 00.49 | 72 | 70.52 | 0.53 | 37 | 70.28 | 0.56 | 42 |
| *Meeting*  *guidelines* | | % |  |  | % |  |  | % |  |
| Meeting guidelines whole week | 64 | 90.14 | 72 | 64 | 85.33 | 37 | 56 | 88.89 | 42 |
| Meeting guidelines week | 66 | 92.96 | 72 | 68 | 90.67 | 37 | 55 | 87.30 | 42 |
| Meeting guidelines weekend | 51 | 71.83 | 72 | 52 | 69.33 | 37 | 49 | 77.78 | 42 |
| Reaching 30 minutes of MVPA in school | 62 | 87.32 | 72 | 65 | 86.67 | 37 | 57 | 90.48 | 42 |
| Reaching 30 minutes of MVPA outside school | 49 | 69.01 | 72 | 47 | 62.67 | 37 | 42 | 66.67 | 42 |

**Follow-Up Data**

|  | **Control (143 children)** | | | **Nonlinear Pedagogy** | |  | **Linear Pedagogy** | |  |
| --- | --- | --- | --- | --- | --- | --- | --- | --- | --- |
| **Variables** | **Mean / Number** | **SD** | **Missing data** | **Mean / Number** | **SD** | **Missing data** | **Mean / Number** | **SD** | **Missing data** |
| Sex (Females) | 83 |  | 0 | 58 |  | 0 | 56 |  | 0 |
| Decimal Age (years) | 6.96 | 0.28 | 1 | 6.94 | 0.30 | 0 | 6.96 | 0.30 | 5 |
| White British | 69 |  | 5 | 54 |  | 9 | 66 |  | 8 |
| SEN (Special Educational Needs) | 17 |  | 0 | 17 |  | 1 | 8 |  | 1 |
| IMD Deprivation Decile (arbitrary units) | 1.73 | 1.51 | 3 | 2.52 | 2.05 | 1 | 1.43 | 1.20 | 4 |
| IOTF SDS BMI (arbitrary units) | 0.23 | 1.46 | 26 | 0.18 | 1.33 | 10 | 0.30 | 1.45 | 9 |
| Participation in school sport events | 0 |  | 0 | 0 |  | 0 | 0 |  | 0 |
| Whole week valid hours (hours) | 15.97 | 1.19 | 84 | 16.31 | 0.80 | 52 | 15.70 | 1.11 | 43 |
| Whole week MVPA (minutes) | 62.59 | 16.00 | 84 | 72.76 | 16.79 | 52 | 66.25 | 18.63 | 43 |
| Whole week Mean ENMO (milligravity) | 56.86 | 12.29 | 84 | 63.64 | 11.74 | 52 | 60.25 | 14.75 | 43 |
| Whole week M60 (milligravity) | 217.30 | 79.03 | 84 | 216.52 | 63.90 | 52 | 220.17 | 85.55 | 43 |
| Weekend valid hours (hours) | 15.82 | 1.59 | 84 | 16.21 | 1.29 | 52 | 15.96 | 1.59 | 43 |
| Weekend MVPA (minutes) | 52.36 | 21.07 | 84 | 65.39 | 23.24 | 52 | 60.95 | 21.99 | 43 |
| Weekend Mean ENMO (milligravity) | 46.56 | 14.43 | 84 | 56.76 | 15.97 | 52 | 53.10 | 15.81 | 43 |
| Weekend M60 (milligravity) | 156.60 | 69.42 | 84 | 189.33 | 93.57 | 52 | 192.97 | 100.25 | 43 |
| In school valid hours (hours) | 5.98 | 0.07 | 84 | 5.95 | 0.11 | 52 | 5.97 | 0.11 | 43 |
| In school MVPA (minutes) | 32.86 | 11.47 | 84 | 37.09 | 9.30 | 52 | 31.85 | 10.39 | 43 |
| In school mean ENMO (milligravity) | 89.16 | 31.39 | 84 | 93.32 | 20.50 | 52 | 86.45 | 26.51 | 43 |
| In school M30 (milligravity) | 159.51 | 80.48 | 84 | 190.96 | 96.44 | 52 | 207.44 | 113.53 | 43 |
| Out of school valid hours (hours) | 7.30 | 1.00 | 84 | 7.52 | 0.64 | 52 | 6.85 | 0.88 | 43 |
| Out of school MVPA (minutes) | 27.13 | 10.09 | 84 | 31.11 | 11.97 | 52 | 27.89 | 11.49 | 43 |
| Out of school mean ENMO (milligravity) | 50.66 | 16.72 | 84 | 56.96 | 17.85 | 52 | 53.19 | 19.40 | 43 |
| Out of school M30 (milligravity) | 191.50 | 80.81 | 84 | 214.30 | 90.65 | 52 | 201.39 | 98.55 | 43 |
| Whole week Temperature (Celsius degrees) | 7.85 | 0.66 | 84 | 7.28 | 0.81 | 52 | 6.15 | 2.05 | 43 |
| Whole week Rainfall (mm water) | 5.56 | 3.47 | 84 | 1.70 | 0.83 | 52 | 3.78 | 2.03 | 43 |
| Whole week percentage of daylight (%) | 42.48 | 6.62 | 84 | 38.53 | 4.52 | 52 | 38.55 | 4.56 | 43 |
| During the week Temperature (Celsius degrees) | 7.47 | 1.01 | 84 | 7.08 | 1.18 | 52 | 5.49 | 2.62 | 43 |
| During the week Rainfall (mm water) | 5.52 | 3.84 | 84 | 1.56 | 1.03 | 52 | 3.44 | 2.10 | 43 |
| During the week percentage of daylight (%) | 42.65 | 0.07 | 84 | 37.58 | 4.64 | 52 | 38.70 | 4.61 | 43 |
| Weekend Temperature (Celsius degrees) | 8.68 | 0.98 | 84 | 7.76 | 1.44 | 52 | 7.82 | 1.21 | 43 |
| Weekend Rainfall (mm water) | 5.62 | 3.30 | 84 | 2.03 | 1.36 | 52 | 4.77 | 3.49 | 43 |
| Weekend percentage of daylight (%) | 42.18 | 6.34 | 84 | 37.47 | 4.37 | 52 | 38.19 | 4.54 | 43 |
| *Meeting*  *guidelines* | | % |  |  | % |  |  | % |  |
| Meeting guidelines whole week | 33 | 55.93 | 84 | 50 | 83.33 | 52 | 38 | 61.29 | 43 |
| Meeting guidelines week | 37 | 62.71 | 84 | 53 | 88.33 | 52 | 40 | 64.52 | 43 |
| Meeting guidelines weekend | 22 | 37.29 | 84 | 30 | 50.00 | 52 | 29 | 46.77 | 43 |
| Reaching 30 minutes of MVPA in school | 31 | 52.54 | 84 | 46 | 76.67 | 52 | 34 | 54.84 | 43 |
| Reaching 30 minutes of MVPA outside school | 23 | 38.98 | 84 | 30 | 50.00 | 52 | 26 | 41.94 | 43 |

**Whole Sample Pooled Data**

| **Row Labels** | **Whole sample** | |  |  |
| --- | --- | --- | --- | --- |
|  | **Mean / Number** | **SD** | **Valid data** | **Missing Data** |
| Sex (Females) | 197 |  | 1080 | 0 |
| Decimal Age (years) | 6.60 | 4.37 | 1056 | 24 |
| White British | 189 |  | 1014 | 66 |
| SEN (Special Educational Needs) | 42 |  | 1074 | 6 |
| IMD Deprivation Decile (arbitrary units) | 1.89 | 1.68 | 1056 | 24 |
| IOTF SDS BMI (arbitrary units) | 0.24 | 1.31 | 956 | 124 |
| Participation in school sport events | 0 |  | 1080 | 0 |
| Whole week valid hours (hours) | 16.10 | 1.12 | 652 | 428 |
| Whole week MVPA (minutes) | 73.74 | 22.21 | 652 | 428 |
| Whole week Mean ENMO (milligravity) | 65.15 | 16.94 | 652 | 428 |
| Whole week M60 (milligravity) | 231.29 | 79.77 | 652 | 428 |
| Weekend valid hours (hours) | 16.00 | 1.56 | 652 | 428 |
| Weekend MVPA (minutes) | 67.84 | 28.89 | 652 | 428 |
| Weekend Mean ENMO (milligravity) | 58.46 | 21.49 | 652 | 428 |
| Weekend M60 (milligravity) | 206.99 | 109.64 | 652 | 428 |
| In school valid hours (hours) | 5.96 | 0.19 | 652 | 428 |
| In school MVPA (minutes) | 36.37 | 11.59 | 652 | 428 |
| In school mean ENMO (milligravity) | 95.33 | 28.29 | 652 | 428 |
| In school M30 (milligravity) | 208.27 | 115.25 | 652 | 428 |
| Out of school valid hours (hours) | 7.36 | 0.91 | 652 | 428 |
| Out of school MVPA (minutes) | 32.14 | 13.81 | 652 | 428 |
| Out of school mean ENMO (milligravity) | 58.48 | 21.97 | 652 | 428 |
| Out of school M30 (milligravity) | 199.69 | 97.44 | 652 | 428 |
| Whole week Temperature (Celsius degrees) | 9.69 | 6.45 | 652 | 428 |
| Whole week Rainfall (mm water) | 2.47 | 2.28 | 652 | 428 |
| Whole week percentage of daylight (%) | 48.02 | 15.9 | 652 | 428 |
| During the week Temperature (Celsius degrees) | 9.64 | 6.77 | 652 | 428 |
| During the week Rainfall (mm water) | 1.97 | 2.37 | 652 | 428 |
| During the week percentage of daylight (%) | 48.01 | 15.91 | 652 | 428 |
| Weekend Temperature (Celsius degrees) | 9.86 | 6.12 | 652 | 428 |
| Weekend Rainfall (mm water) | 3.34 | 2.88 | 652 | 428 |
| Weekend percentage of daylight (%) | 48.03 | 15.89 | 652 | 428 |
| *Meeting guidelines* | | % |  |  |
| Meeting guidelines whole week | 474 | 72.70 | 652 | 428 |
| Meeting guidelines week | 505 | 77.45 | 652 | 428 |
| Meeting guidelines weekend | 367 | 56.29 | 652 | 428 |
| Reaching 30 minutes of MVPA in school | 453 | 69.48 | 652 | 428 |
| Reaching 30 minutes of MVPA outside school | 343 | 52.61 | 652 | 428 |

MVPA: Moderate to vigorous physical activity; ENMO: Euclidean norm minus one; M60: minimum acceleration value in the most active hour; M30: minimum acceleration value in the most active half hour; SD: standard error; IMD: index of neighbourhood multiple deprivation decile; IOTF SDS BMI: International Obesity Task Force standardised Body Mass Index.
