# Supplementary material for: Effect of Linear and Nonlinear Pedagogy Physical Education Interventions on Children’s Physical Activity: A Cluster Randomized Controlled Trial (SAMPLE-PE)
Source: Children (Basel). 2021 Jan 15;8(1):49. doi: 10.3390/children8010049 (PMC7830495; doi:10.3390/children8010049)
Supplement: Supplementary file 1 [file children-08-00049-s001.zip › children-1040334-supplementary/children-1040334-Supplementry material Table S4 - Intention to treat analysis.docx]

**Table S4.** Intention to treat analysis.

| **Whole Week Physical Activity** | | | | | | | | | |
| --- | --- | --- | --- | --- | --- | --- | --- | --- | --- |
|  | **MVPA** |  |  | **Mean ENMO** | |  | **M60** |  |  |
| **Predictors** | **Estimate** | **Std. error** | **p-value** | **estimate** | **Std. error** | **p-value** | **Estimate** | **Std error** | **p-value** |
| (Intercept) | -18.75 | 24.75 | 0.454 | -7.889 | 14.908 | 0.598 | 128.752 | 79.585 | 0.111 |
| Time [T1 Vs T0] | -4.00 | 3.90 | 0.318 | -2.997 | 3.088 | 0.345 | -12.608 | 11.046 | 0.262 |
| Time [T2 Vs T0] | -6.59 | 4.60 | 0.163 | -5.604 | 2.940 | 0.062 | -13.141 | 12.777 | 0.306 |
| Group [NLP Vs Control] | 2.14 | 3.22 | 0.509 | 0.795 | 2.332 | 0.734 | -14.335 | 12.029 | 0.238 |
| Group [LP Vs Control] | 3.16 | 2.86 | 0.269 | 1.700 | 2.391 | 0.479 | -3.962 | 11.166 | 0.723 |
| Decimal Age | 5.19 | 3.45 | 0.141 | 5.084 | 2.116 | 0.017 | 10.603 | 11.332 | 0.353 |
| Sex | -12.10 | 2.07 | <0.001 | -9.335 | 1.446 | <0.001 | -54.850 | 7.887 | <0.001 |
| IOTF SDS BMI | -0.72 | 0.84 | 0.403 | -0.941 | 0.596 | 0.124 | -5.407 | 2.671 | 0.049 |
| Special educational needs | -1.20 | 3.90 | 0.760 | -1.282 | 2.176 | 0.556 | -15.054 | 11.926 | 0.209 |
| Index of multiple deprivation | -0.29 | 0.60 | 0.629 | -0.196 | 0.515 | 0.705 | -0.179 | 2.360 | 0.940 |
| Ethnicity code | 1.77 | 1.97 | 0.370 | 4.304 | 1.608 | 0.009 | 22.100 | 9.184 | 0.022 |
| Sport events | 1.49 | 4.00 | 0.711 | 2.718 | 3.307 | 0.417 | 13.902 | 13.505 | 0.308 |
| Mean rainfall (mm rain) | -0.94 | 0.43 | 0.043 | -0.849 | 0.255 | 0.002 | -2.648 | 1.005 | 0.010 |
| Mean Temperature (Celsius degrees) | 0.25 | 0.30 | 0.414 | 0.244 | 0.246 | 0.333 | 2.053 | 1.106 | 0.076 |
| Daylight (% of day duration) | 0.48 | 0.15 | 0.006 | 0.325 | 0.099 | 0.003 | 0.710 | 0.410 | 0.094 |
| Valid wear time | 2.78 | 0.71 | 0.001 | 1.894 | 0.523 | 0.001 | 1.483 | 2.364 | 0.535 |
| Time [T1] * Group [NLP] Vs Control | -2.62 | 3.17 | 0.414 | -1.881 | 2.652 | 0.483 | -1.805 | 10.466 | 0.864 |
| Time [T2] * Group [NLP] Vs Control | 1.57 | 3.75 | 0.680 | 0.402 | 2.448 | 0.870 | 3.156 | 11.981 | 0.794 |
| Time [T1] * Group [LP] Vs Control | -0.64 | 4.04 | 0.876 | -0.936 | 2.850 | 0.743 | -0.071 | 14.383 | 0.996 |
| Time [T2] * Group [LP] Vs Control | -2.07 | 3.33 | 0.538 | -2.204 | 2.539 | 0.390 | -1.692 | 11.085 | 0.879 |
| σ2 | 160.87 |  |  | 100.49 |  |  | 1948.89 |  |  |
| τ002 Children | 213.53 |  |  | 110.92 |  |  | 3008.79 |  |  |
| Intraclass correlation coefficient | 0.57 |  |  | 0.52 |  |  | 0.61 |  |  |
| Number of children | 360 |  |  | 360 |  |  | 360 |  |  |
| Observations | 1080 |  |  | 1080 |  |  | 1080 |  |  |
| Marginal R2 / Conditional R2 | 0.24/0.67 |  |  | 0.26/0.65 |  |  | 0.22/0.69 |  |  |

**Weekend Physical Activity**

|  | **MVPA** |  |  | **Mean ENMO** | |  | **M60** |  |  |
| --- | --- | --- | --- | --- | --- | --- | --- | --- | --- |
| **Predictors** | **Estimate** | **Std. error** | **p-value** | **estimate** | **Std. error** | **p-value** | **Estimate** | **Std error** | **p-value** |
| (Intercept) | -60.06 | 25.31 | 0.019 | -25.92 | 20.29 | 0.205 | 15.78 | 104.47 | 0.880 |
| Time [T1 Vs T0] | -8.63 | 4.51 | 0.063 | -5.38 | 3.65 | 0.152 | -21.81 | 13.82 | 0.118 |
| Time [T2 Vs T0] | -13.39 | 5.11 | 0.010 | -10.31 | 4.46 | 0.026 | -28.46 | 18.82 | 0.132 |
| Group [NLP Vs Control] | 0.73 | 4.00 | 0.855 | -0.23 | 3.42 | 0.946 | -4.08 | 15.39 | 0.791 |
| Group [LP Vs Control] | 3.47 | 4.74 | 0.468 | 2.90 | 3.35 | 0.389 | 20.37 | 15.36 | 0.186 |
| Decimal Age | 9.52 | 3.97 | 0.018 | 6.69 | 3.43 | 0.058 | 14.95 | 16.10 | 0.354 |
| Sex | -10.12 | 3.42 | 0.006 | -6.91 | 2.21 | 0.003 | -36.94 | 12.47 | 0.004 |
| IOTF SDS BMI | -0.90 | 1.05 | 0.396 | -1.20 | 0.81 | 0.149 | -6.11 | 3.66 | 0.099 |
| Special educational needs | -4.41 | 4.27 | 0.304 | -3.30 | 3.13 | 0.294 | -20.11 | 17.64 | 0.256 |
| Index of multiple deprivation | 0.14 | 0.85 | 0.869 | -0.17 | 0.58 | 0.767 | 3.43 | 3.36 | 0.309 |
| Ethnicity code | 7.29 | 2.86 | 0.013 | 8.15 | 2.17 | <0.001 | 47.87 | 10.93 | <0.001 |
| Sport events | -4.56 | 5.44 | 0.404 | -2.71 | 4.34 | 0.535 | -18.43 | 18.21 | 0.314 |
| Mean rainfall (mm rain) | -0.79 | 0.43 | 0.080 | -0.73 | 0.33 | 0.038 | -2.80 | 1.28 | 0.034 |
| Mean Temperature (Celsius degrees) | 0.40 | 0.38 | 0.298 | 0.29 | 0.31 | 0.350 | 0.77 | 1.22 | 0.529 |
| Daylight (% of day duration) | 0.51 | 0.17 | 0.006 | 0.34 | 0.14 | 0.030 | 1.00 | 0.48 | 0.044 |
| Valid wear time | 3.12 | 0.65 | <0.001 | 1.86 | 0.44 | <0.001 | 3.23 | 1.99 | 0.108 |
| Time [T1] * Group [NLP] Vs Control | -2.50 | 4.68 | 0.595 | -0.75 | 4.26 | 0.861 | 7.55 | 14.69 | 0.608 |
| Time [T2] * Group [NLP] Vs Control | 1.67 | 5.39 | 0.758 | 2.74 | 4.16 | 0.515 | 7.61 | 14.86 | 0.610 |
| Time [T1] * Group [LP] Vs Control | 0.64 | 4.88 | 0.897 | -0.81 | 4.02 | 0.841 | 4.76 | 18.96 | 0.803 |
| Time [T2] * Group [LP] Vs Control | -3.91 | 4.87 | 0.426 | -1.74 | 4.18 | 0.680 | -13.69 | 14.70 | 0.355 |
| σ2 | 399.93 |  |  | 228.19 |  |  | 4254.6 |  |  |
| τ002 Children | 323.41 |  |  | 174.87 |  |  | 7163.9 |  |  |
| Intraclass correlation coefficient | 0.45 |  |  | 0.43 |  |  | 0.6 |  |  |
| Number of children | 360 |  |  | 360 |  |  | 360 |  |  |
| Observations | 1080 |  |  | 1080 |  |  | 1080 |  |  |
| Marginal R2 / Conditional R2 | 0.17/0.54 |  |  | 0.18/0.53 |  |  | 0.11/0.67 |  |  |

**In School Physical Activity**

|  | **MVPA** |  |  | **Mean ENMO** | |  | **M30** |  |  |
| --- | --- | --- | --- | --- | --- | --- | --- | --- | --- |
| **Predictors** | **Estimate** | **Std. error** | **p-value** | **estimate** | **Std. error** | **p-value** | **Estimate** | **Std error** | **p-value** |
| (Intercept) | 16.46 | 11.88 | 0.172 | 73.88 | 29.84 | 0.018 | 263.559 | 121.715 | 0.035 |
| Time [T1 Vs T0] | -1.38 | 1.60 | 0.396 | -6.05 | 4.25 | 0.166 | -12.287 | 14.541 | 0.402 |
| Time [T2 Vs T0] | -3.36 | 1.73 | 0.053 | -4.06 | 4.58 | 0.379 | -12.443 | 19.191 | 0.519 |
| Group [NLP Vs Control] | -0.18 | 2.25 | 0.936 | -2.32 | 5.51 | 0.674 | -18.288 | 22.233 | 0.411 |
| Group [LP Vs Control] | -2.38 | 2.23 | 0.288 | -8.91 | 5.60 | 0.112 | -45.447 | 22.58 | 0.045 |
| Decimal Age | 2.18 | 1.48 | 0.142 | 2.09 | 4.00 | 0.605 | 10.73 | 16.713 | 0.523 |
| Sex | -7.29 | 1.06 | <0.001 | -20.28 | 2.55 | <0.001 | -92.133 | 11.246 | <0.001 |
| IOTF SDS BMI | -0.13 | 0.41 | 0.756 | -1.24 | 0.75 | 0.098 | -5.933 | 3.367 | 0.081 |
| Special educational needs | 2.00 | 1.67 | 0.235 | 4.01 | 3.92 | 0.308 | 1.874 | 18.093 | 0.918 |
| Index of multiple deprivation | -0.16 | 0.32 | 0.631 | -1.09 | 0.77 | 0.159 | -3.377 | 3.199 | 0.292 |
| Ethnicity code | -0.86 | 1.28 | 0.506 | 0.52 | 2.53 | 0.837 | -6.843 | 12.833 | 0.595 |
| Sport events | 4.95 | 2.30 | 0.039 | 15.23 | 5.38 | 0.007 | 61.307 | 19.901 | 0.003 |
| Mean rainfall (mm rain) | -0.34 | 0.15 | 0.022 | -1.08 | 0.46 | 0.024 | -1.045 | 1.61 | 0.519 |
| Mean Temperature (Celsius degrees) | 0.22 | 0.14 | 0.114 | 1.00 | 0.31 | 0.002 | 4.839 | 1.405 | 0.002 |
| Daylight (% of day duration) | 0.12 | 0.06 | 0.071 | 0.25 | 0.15 | 0.097 | -0.074 | 0.569 | 0.897 |
| Valid wear time | 0.90 | 1.07 | 0.411 | 1.40 | 2.51 | 0.582 | 4.623 | 10.613 | 0.666 |
| Time [T1] * Group [NLP] Vs Control | -1.56 | 1.55 | 0.318 | -3.29 | 3.57 | 0.358 | -14.936 | 13.151 | 0.257 |
| Time [T2] * Group [NLP] Vs Control | 2.23 | 1.57 | 0.162 | 1.45 | 5.19 | 0.783 | -3.185 | 15.374 | 0.837 |
| Time [T1] * Group [LP] Vs Control | 0.81 | 2.27 | 0.724 | 0.71 | 5.02 | 0.887 | -5.437 | 18.36 | 0.768 |
| Time [T2] * Group [LP] Vs Control | 0.39 | 1.48 | 0.792 | 2.62 | 3.72 | 0.482 | 2.341 | 14.128 | 0.869 |
| σ2 | 43.63 |  |  | 284.84 |  |  | 4148.77 |  |  |
| τ002 Children | 51.02 |  |  | 267.33 |  |  | 6073.31 |  |  |
| τ002 Class | 7.40 |  |  | 48.88 |  |  | 841.46 |  |  |
| Intraclass correlation coefficient | 0.57 |  |  | 0.65 |  |  | 0.63 |  |  |
| Number of children | 360 |  |  | 360 |  |  | 360 |  |  |
| N classes | 18 |  |  | 18 |  |  | 18 |  |  |
| Observations | 1080 |  |  | 1080 |  |  | 1080 |  |  |
| Marginal R2 / Conditional R2 | 0.23/0.67 |  |  | 0.27/0.65 |  |  | 0.23/0.71 |  |  |
|  |  |  |  |  |  |  |  |  |  |

**Out of School Physical Activity from 15:00 to 23:00**

|  | **MVPA** |  |  | **Mean ENMO** | |  | **M30** |  |  |
| --- | --- | --- | --- | --- | --- | --- | --- | --- | --- |
| **Predictors** | **Estimate** | **Std. error** | **p-value** | **estimate** | **Std. error** | **p-value** | **Estimate** | **Std error** | **p-value** |
| (Intercept) | -18.90 | 11.54 | 0.103 | -14.94 | 25.46 | 0.562 | -71.88 | 78.12 | 0.358 |
| Time [T1 Vs T0] | -0.41 | 2.26 | 0.858 | -0.70 | 3.36 | 0.836 | 4.82 | 14.82 | 0.748 |
| Time [T2 Vs T0] | -1.58 | 2.42 | 0.516 | -3.27 | 4.03 | 0.420 | 6.96 | 16.13 | 0.667 |
| Group [NLP Vs Control] | 2.01 | 2.03 | 0.324 | 2.16 | 3.21 | 0.503 | 0.35 | 12.82 | 0.978 |
| Group [LP Vs Control] | 4.19 | 2.17 | 0.058 | 5.90 | 3.18 | 0.066 | 8.28 | 12.63 | 0.513 |
| Decimal Age | 2.83 | 1.90 | 0.140 | 4.10 | 3.40 | 0.233 | 14.14 | 12.19 | 0.248 |
| Sex | -3.03 | 1.29 | 0.021 | -5.92 | 1.87 | 0.002 | -37.50 | 7.92 | <0.001 |
| IOTF SDS BMI | -0.40 | 0.42 | 0.342 | -0.69 | 0.92 | 0.459 | -4.16 | 3.32 | 0.218 |
| Special educational needs | -4.11 | 2.12 | 0.057 | -6.38 | 2.93 | 0.031 | -27.87 | 13.75 | 0.046 |
| Index of multiple deprivation | -0.05 | 0.34 | 0.882 | -0.06 | 0.65 | 0.930 | 0.66 | 2.52 | 0.794 |
| Ethnicity code | 2.19 | 1.54 | 0.164 | 5.14 | 2.43 | 0.042 | 28.25 | 10.04 | 0.008 |
| Sport events | 1.87 | 2.49 | 0.454 | 2.78 | 4.93 | 0.577 | 18.55 | 15.62 | 0.237 |
| Mean rainfall (mm rain) | -0.53 | 0.22 | 0.017 | -0.74 | 0.38 | 0.058 | -1.48 | 1.56 | 0.351 |
| Mean Temperature (Celsius degrees) | -0.13 | 0.19 | 0.502 | -0.21 | 0.26 | 0.439 | 0.15 | 1.31 | 0.912 |
| Daylight (% of day duration) | 0.35 | 0.08 | <0.001 | 0.52 | 0.14 | 0.001 | 2.38 | 0.55 | <0.001 |
| Valid wear time | 1.89 | 0.42 | <0.001 | 2.61 | 0.79 | 0.002 | 6.52 | 2.87 | 0.029 |
| Time [T1] * Group [NLP] Vs Control | -2.09 | 2.11 | 0.326 | -1.58 | 3.20 | 0.623 | 3.24 | 13.46 | 0.811 |
| Time [T2] * Group [NLP] Vs Control | -0.28 | 2.27 | 0.902 | 0.49 | 3.83 | 0.899 | 10.71 | 13.04 | 0.413 |
| Time [T1] * Group [LP] Vs Control | -4.17 | 2.69 | 0.126 | -5.84 | 4.78 | 0.228 | -15.90 | 16.16 | 0.327 |
| Time [T2] * Group [LP] Vs Control | -3.89 | 2.19 | 0.079 | -4.30 | 3.73 | 0.253 | 0.11 | 12.67 | 0.993 |
| σ2 | 92.42 |  |  | 243.02 |  |  | 3437.31 |  |  |
| τ002 Children | 69.58 |  |  | 165.69 |  |  | 3357.13 |  |  |
| Intraclass correlation coefficient | 0.43 |  |  | 0.41 |  |  | 0.50 |  |  |
| Number of children | 360 |  |  | 360 |  |  | 360 |  |  |
| Observations | 1080 |  |  | 1080 |  |  | 1080 |  |  |
| Marginal R2 / Conditional R2 | 0.16/0.52 |  |  | 0.15/0.50 |  |  | 0.23/0.61 |  |  |

MVPA: Moderate to vigorous physical activity; ENMO: Euclidean norm minus one; M60: minimum acceleration value in the most active hour; M30: minimum acceleration value in the most active half hour; Std. error: standard error; T0: Baseline; T1: Post Intervention T2: Follow-up; NLP: Nonlinear Pedagogy group; LP: Linear Pedagogy group; IOTF SDS BMI: International Obesity Task Force standardised Body Mass Index, σ2: Intercept variance; τ002: Random factor variance.
