# Supplementary material for: Effect of Linear and Nonlinear Pedagogy Physical Education Interventions on Children’s Physical Activity: A Cluster Randomized Controlled Trial (SAMPLE-PE)
Source: Children (Basel). 2021 Jan 15;8(1):49. doi: 10.3390/children8010049 (PMC7830495; doi:10.3390/children8010049)
Supplement: Supplementary file 1 [file children-08-00049-s001.zip › children-1040334-supplementary/children-1040334-Supplementry material Table S5 - Complete cases analysis.docx]

**Table S5.** Complete cases analysis.

| **Whole week Physical Activity** | | | | | | | | | |
| --- | --- | --- | --- | --- | --- | --- | --- | --- | --- |
|  | **MVPA** |  |  | **Mean ENMO** | |  | **M60** |  |  |
| **Predictors** | **Estimate** | **Std. error** | **p-value** | **estimate** | **Std. error** | **p-value** | **Estimate** | **Std. error** | **p-value** |
| (Intercept) | -56.23 | 23.95 | 0.019 | -19.91 | 17.97 | 0.268 | 51.58 | 88.71 | 0.561 |
| Time [T1 Vs T0] | -12.71 | 11.66 | 0.276 | -5.78 | 9.00 | 0.521 | -55.25 | 43.83 | 0.207 |
| Time [T2 Vs T0] | -15.98 | 4.91 | 0.001 | -10.75 | 3.74 | 0.004 | -21.11 | 18.34 | 0.25 |
| Group [NLP Vs Control] | 1.19 | 3.40 | 0.726 | 0.28 | 2.59 | 0.915 | -15.45 | 12.68 | 0.223 |
| Group [LP Vs CG] | 2.65 | 3.32 | 0.425 | 1.77 | 2.53 | 0.483 | 0.96 | 12.39 | 0.938 |
| Decimal Age | 10.50 | 3.42 | 0.002 | 7.10 | 2.54 | 0.005 | 16.64 | 12.60 | 0.187 |
| Sex | -11.93 | 2.05 | <0.001 | -10.24 | 1.52 | <0.001 | -60.77 | 7.54 | <0.001 |
| IOTF SDS BMI | -0.80 | 0.68 | 0.242 | -1.01 | 0.51 | 0.05 | -6.35 | 2.54 | 0.012 |
| Special educational needs | -4.51 | 3.21 | 0.16 | -3.12 | 2.39 | 0.192 | -20.37 | 11.83 | 0.085 |
| Index of multiple deprivation | -0.13 | 0.61 | 0.827 | -0.34 | 0.45 | 0.449 | -0.22 | 2.24 | 0.92 |
| Ethnicity code | 2.03 | 2.06 | 0.324 | 3.70 | 1.53 | 0.015 | 22.60 | 7.58 | 0.003 |
| Sport events | 4.15 | 4.69 | 0.377 | 3.94 | 3.66 | 0.281 | 19.58 | 17.72 | 0.269 |
| Mean rainfall (mm rain) | -0.77 | 0.43 | 0.073 | -0.52 | 0.33 | 0.116 | -3.83 | 1.61 | 0.017 |
| Mean Temperature (Celsius degrees) | 0.37 | 0.45 | 0.415 | 0.25 | 0.35 | 0.477 | 2.36 | 1.69 | 0.162 |
| Daylight (% of day duration) | 0.62 | 0.23 | 0.008 | 0.39 | 0.18 | 0.028 | 1.79 | 0.87 | 0.039 |
| Valid wear time | 2.79 | 0.67 | <0.001 | 1.77 | 0.52 | 0.001 | 1.85 | 2.52 | 0.462 |
| Time [T1] * Group [NLP] Vs Control | -2.02 | 3.71 | 0.587 | -0.32 | 2.90 | 0.913 | 0.28 | 14.03 | 0.984 |
| Time [T2] * Group [NLP] Vs Control | 5.73 | 4.35 | 0.188 | 4.52 | 3.40 | 0.183 | 3.12 | 16.44 | 0.849 |
| Time [T1] * Group [LP] Vs Control | -1.63 | 4.94 | 0.742 | -1.65 | 3.86 | 0.668 | -6.98 | 18.67 | 0.708 |
| Time [T2] * Group [LP] Vs Control | -1.22 | 4.04 | 0.762 | -0.80 | 3.16 | 0.799 | -10.63 | 15.28 | 0.487 |
| σ2 | 156.34 |  |  | 97.2 |  |  | 2254.68 |  |  |
| τ002 Children | 180.15 |  |  | 93.44 |  |  | 2376.08 |  |  |
| Intraclass correlation coefficient | 0.54 |  |  | 0.49 |  |  | 0.51 |  |  |
| Number of children | 274 |  |  | 274 |  |  | 274 |  |  |
| Observations | 575 |  |  | 575 |  |  | 575 |  |  |
| Marginal R2 / Conditional R2 | 0.31/0.68 |  |  | 0.34/0.66 |  |  | 0.29/0.65 |  |  |

**Weekend Physical Activity**

|  | **MVPA** |  |  | **Mean ENMO** | |  | **M60** |  |  |
| --- | --- | --- | --- | --- | --- | --- | --- | --- | --- |
| **Predictors** | **Estimate** | **Std. error** | **p-value** | **estimate** | **Std. error** | **p-value** | **Estimate** | **Std error** | **p-value** |
| (Intercept) | -108.11 | 30.73 | <0.001 | -66.22 | 22.79 | 0.004 | -172.44 | 121.64 | 0.156 |
| Time [T1 Vs T0] | -32.50 | 15.81 | 0.04 | -22.38 | 11.86 | 0.059 | -142.88 | 62.54 | 0.022 |
| Time [T2 Vs T0] | -29.06 | 6.89 | <0.001 | -22.31 | 5.16 | <0.001 | -87.39 | 27.26 | 0.001 |
| Group [NLP Vs Control] | 0.33 | 4.77 | 0.945 | -0.34 | 3.57 | 0.924 | -6.60 | 18.89 | 0.727 |
| Group [LP Vs Control] | 6.66 | 4.72 | 0.158 | 5.19 | 3.53 | 0.142 | 28.66 | 18.69 | 0.125 |
| Decimal Age | 14.87 | 4.44 | 0.001 | 11.40 | 3.28 | 0.001 | 30.78 | 17.59 | 0.08 |
| Sex | -10.15 | 2.66 | <0.001 | -7.35 | 1.97 | <0.001 | -37.05 | 10.54 | <0.001 |
| IOTF SDS BMI | -1.13 | 0.93 | 0.224 | -1.32 | 0.69 | 0.057 | -7.48 | 3.69 | 0.043 |
| Special educational needs | -7.11 | 4.21 | 0.092 | -5.08 | 3.12 | 0.103 | -28.74 | 16.69 | 0.085 |
| Index of multiple deprivation | 0.11 | 0.79 | 0.888 | -0.19 | 0.58 | 0.748 | 0.36 | 3.12 | 0.908 |
| Ethnicity code | 6.02 | 2.68 | 0.025 | 7.47 | 1.98 | <0.001 | 52.29 | 10.62 | <0.001 |
| Sport events | -9.44 | 7.04 | 0.18 | -7.38 | 5.32 | 0.165 | -51.98 | 27.84 | 0.062 |
| Mean rainfall (mm rain) | -1.12 | 0.45 | 0.012 | -0.79 | 0.34 | 0.02 | -4.27 | 1.77 | 0.016 |
| Mean Temperature (Celsius degrees) | 1.08 | 0.56 | 0.052 | 0.79 | 0.42 | 0.06 | 4.23 | 2.21 | 0.055 |
| Daylight (% of day duration) | 0.86 | 0.34 | 0.012 | 0.57 | 0.26 | 0.026 | 3.02 | 1.35 | 0.026 |
| Valid wear time | 3.23 | 0.68 | <0.001 | 1.99 | 0.51 | <0.001 | 4.15 | 2.68 | 0.122 |
| Time [T1] * Group [NLP] Vs Control | -1.18 | 5.86 | 0.841 | 1.97 | 4.44 | 0.656 | 19.29 | 23.17 | 0.405 |
| Time [T2] * Group [NLP] Vs Control | 9.41 | 6.57 | 0.152 | 8.71 | 4.97 | 0.08 | 33.42 | 25.96 | 0.198 |
| Time [T1] * Group [LP] Vs Control | 0.88 | 7.40 | 0.905 | -0.28 | 5.60 | 0.959 | 12.70 | 29.26 | 0.664 |
| Time [T2] * Group [LP] Vs Control | -0.88 | 5.87 | 0.881 | -0.91 | 4.44 | 0.838 | -2.41 | 23.19 | 0.917 |
| σ2 | 403.96 |  |  | 233.58 |  |  | 6310.18 |  |  |
| τ002 Children | 234.45 |  |  | 121.24 |  |  | 3685.88 |  |  |
| Intraclass correlation coefficient | 0.37 |  |  | 0.34 |  |  | 0.37 |  |  |
| Number of children | 274 |  |  | 274 |  |  | 274 |  |  |
| Observations | 575 |  |  | 575 |  |  | 575 |  |  |
| Marginal R2 / Conditional R2 | 0.23/0.51 |  |  | 0.24/0.50 |  |  | 0.17/0.48 |  |  |

**In school Physical Activity**

|  | **MVPA** |  |  | **Mean ENMO** | |  | **M30** |  |  |
| --- | --- | --- | --- | --- | --- | --- | --- | --- | --- |
| **Predictors** | **Estimate** | **Std. error** | **p-value** | **estimate** | **Std. error** | **p-value** | **Estimate** | **Std error** | **p-value** |
| (Intercept) | 10.99 | 15.53 | 0.479 | 28.90 | 38.50 | 0.453 | 51.86 | 167.19 | 0.756 |
| Time [T1 Vs T0] | 8.29 | 5.62 | 0.14 | 16.69 | 14.44 | 0.248 | -12.33 | 64.07 | 0.847 |
| Time [T2 Vs T0] | -5.59 | 2.42 | 0.021 | -9.32 | 6.05 | 0.123 | -3.78 | 26.42 | 0.886 |
| Group [NLP Vs Control] | -2.35 | 2.80 | 0.403 | -5.96 | 6.81 | 0.381 | -20.90 | 29.41 | 0.477 |
| Group [LP Vs Control] | -5.37 | 2.84 | 0.059 | -12.25 | 6.90 | 0.076 | -51.34 | 29.81 | 0.085 |
| Decimal Age | 2.85 | 1.66 | 0.086 | 5.96 | 3.98 | 0.134 | 19.71 | 16.93 | 0.244 |
| Sex | -7.47 | 0.98 | <0.001 | -20.54 | 2.36 | <0.001 | -94.09 | 10.04 | <0.001 |
| IOTF SDS BMI | -0.07 | 0.33 | 0.844 | -0.78 | 0.81 | 0.333 | -4.56 | 3.49 | 0.191 |
| Special educational needs | 0.43 | 1.58 | 0.783 | 2.12 | 3.81 | 0.577 | 3.13 | 16.24 | 0.847 |
| Index of multiple deprivation | 0.01 | 0.30 | 0.975 | -0.61 | 0.72 | 0.391 | -1.47 | 3.05 | 0.629 |
| Ethnicity code | -0.34 | 1.11 | 0.757 | 1.77 | 2.66 | 0.506 | 2.39 | 11.33 | 0.833 |
| Sport events | 7.55 | 2.41 | 0.002 | 21.51 | 6.26 | 0.001 | 91.51 | 27.93 | 0.001 |
| Mean rainfall (mm rain) | -0.07 | 0.22 | 0.733 | -0.63 | 0.56 | 0.262 | -4.47 | 2.48 | 0.071 |
| Mean Temperature (Celsius degrees) | 0.04 | 0.20 | 0.835 | 0.51 | 0.52 | 0.331 | 5.71 | 2.33 | 0.014 |
| Daylight (% of day duration) | -0.12 | 0.12 | 0.327 | -0.26 | 0.31 | 0.398 | -0.55 | 1.37 | 0.686 |
| Valid wear time | 2.91 | 1.89 | 0.124 | 8.62 | 4.77 | 0.071 | 34.30 | 20.96 | 0.102 |
| Time [T1] * Group [NLP] Vs Control | 0.16 | 1.78 | 0.93 | -0.86 | 4.61 | 0.852 | -14.68 | 20.57 | 0.475 |
| Time [T2] * Group [NLP] Vs Control | 5.18 | 2.11 | 0.014 | 7.42 | 5.46 | 0.174 | -25.53 | 24.34 | 0.294 |
| Time [T1] * Group [LP] Vs Control | 1.98 | 2.56 | 0.439 | 1.33 | 6.64 | 0.841 | -4.73 | 29.59 | 0.873 |
| Time [T2] * Group [LP] Vs Control | 2.34 | 2.01 | 0.244 | 5.08 | 5.20 | 0.329 | -6.74 | 23.19 | 0.771 |
| σ2 | 38.99 |  |  | 267.81 |  |  | 5393.32 |  |  |
| τ002 Children | 38.22 |  |  | 198.8 |  |  | 3337.95 |  |  |
| τ002 Class | 15.49 |  |  | 88.34 |  |  | 1628.16 |  |  |
| Intraclass correlation coefficient | 0.58 |  |  | 0.52 |  |  | 0.48 |  |  |
| Number of children | 274 |  |  | 274 |  |  | 274 |  |  |
| N classes | 18 |  |  | 18 |  |  | 18 |  |  |
| Observations | 575 |  |  | 575 |  |  | 575 |  |  |
| Marginal R2 / Conditional R2 | 0.29/0.70 |  |  | 0.33/0.68 |  |  | 0.30/0.63 |  |  |

**Out of School Physical Activity from 15:00 to 23:00**

|  | **MVPA** |  |  | **Mean ENMO** | |  | **M30** |  |  |
| --- | --- | --- | --- | --- | --- | --- | --- | --- | --- |
| **Predictors** | **Estimate** | **Std. error** | **p-value** | **estimate** | **Std. error** | **p-value** | **Estimate** | **Std error** | **p-value** |
| (Intercept) | -31.69 | 14.94 | 0.034 | -30.84 | 23.75 | 0.194 | -180.01 | 97.86 | 0.066 |
| Time [T1 Vs T0] | -1.65 | 7.68 | 0.830 | 0.05 | 12.18 | 0.997 | -38.47 | 50.87 | 0.450 |
| Time [T2 Vs T0] | -3.83 | 3.34 | 0.251 | -4.10 | 5.30 | 0.439 | 29.67 | 22.01 | 0.178 |
| Group [NLP Vs Control] | 2.56 | 2.30 | 0.266 | 4.59 | 3.66 | 0.210 | 13.41 | 15.19 | 0.377 |
| Group [LP Vs Control] | 4.85 | 2.26 | 0.032 | 8.45 | 3.59 | 0.019 | 30.66 | 14.90 | 0.040 |
| Decimal Age | 4.76 | 2.23 | 0.033 | 6.47 | 3.55 | 0.068 | 17.62 | 14.57 | 0.226 |
| Sex | -3.00 | 1.33 | 0.024 | -5.67 | 2.12 | 0.008 | -42.02 | 8.71 | <0.001 |
| IOTF SDS BMI | -0.22 | 0.46 | 0.635 | -0.57 | 0.74 | 0.435 | -4.79 | 3.04 | 0.115 |
| Special educational needs | -4.51 | 2.11 | 0.032 | -7.12 | 3.35 | 0.034 | -31.83 | 13.77 | 0.021 |
| Index of multiple deprivation | -0.11 | 0.40 | 0.781 | -0.26 | 0.63 | 0.679 | 1.01 | 2.59 | 0.697 |
| Ethnicity code | 1.03 | 1.34 | 0.445 | 3.51 | 2.14 | 0.100 | 27.66 | 8.77 | 0.002 |
| Sport events | 5.20 | 3.44 | 0.131 | 8.40 | 5.46 | 0.124 | 31.02 | 22.87 | 0.175 |
| Mean rainfall (mm rain) | -0.44 | 0.29 | 0.135 | -0.68 | 0.47 | 0.143 | -3.36 | 1.95 | 0.085 |
| Mean Temperature (Celsius degrees) | -0.20 | 0.29 | 0.480 | -0.50 | 0.45 | 0.272 | -0.03 | 1.90 | 0.987 |
| Daylight (% of day duration) | 0.41 | 0.16 | 0.010 | 0.64 | 0.25 | 0.011 | 4.26 | 1.06 | <0.001 |
| Valid wear time | 1.81 | 0.47 | <0.001 | 2.39 | 0.75 | 0.001 | 7.40 | 3.13 | 0.018 |
| Time [T1] * Group [NLP] Vs Control | -2.61 | 2.64 | 0.323 | -3.37 | 4.19 | 0.421 | -4.48 | 17.59 | 0.799 |
| Time [T2] * Group [NLP] Vs Control | 0.61 | 3.07 | 0.844 | 0.52 | 4.87 | 0.916 | 6.64 | 20.43 | 0.745 |
| Time [T1] * Group [LP] Vs Control | -7.74 | 3.71 | 0.037 | -12.24 | 5.89 | 0.038 | -48.03 | 24.70 | 0.052 |
| Time [T2] * Group [LP] Vs Control | -4.41 | 2.94 | 0.134 | -7.44 | 4.66 | 0.111 | -23.25 | 19.58 | 0.235 |
| σ2 | 90.96 |  |  | 228.21 |  |  | 4057.75 |  |  |
| τ002 Children | 64.3 |  |  | 163.66 |  |  | 2652.47 |  |  |
| Intraclass correlation coefficient | 0.41 |  |  | 0.42 |  |  | 0.4 |  |  |
| Number of children | 274 |  |  | 274 |  |  | 274 |  |  |
| Observations | 575 |  |  | 575 |  |  | 575 |  |  |
| Marginal R2 / Conditional R2 | 0.18/0.52 |  |  | 0.17/0.52 |  |  | 0.32/0.59 |  |  |

MVPA: Moderate to vigorous physical activity; ENMO: Euclidean norm minus one; M60: minimum acceleration value in the most active hour; M30: minimum acceleration value in the most active half hour; Std. error: standard error; T0: Baseline; T1: Post Intervention T2: Follow-up; NLP: Nonlinear Pedagogy group; LP: Linear Pedagogy group; IOTF SDS BMI: International Obesity Task Force standardised Body Mass Index, σ2: Intercept variance; τ002: Random factor variance.
